# Clinical and echocardiographic response to volume expansion in hypotensive preterm infants: a pilot observational study

**DOI:** 10.3389/fped.2026.1749902

**Published:** 2026-03-04

**Authors:** Océane Lalin, Jean-Marc Jellimann, Jean-Michel Hascoet

**Affiliations:** 1 Neonatology Department, Maternité Régionale Universitaire—CHRU Nancy, Nancy, France; 2 DevAH 3450, Université de Lorraine, Nancy, France

**Keywords:** hemodynamic impairment, neonate, preterm, ultrasound, volume expansion

## Abstract

**Objective:**

To describe the short-term clinical and echocardiographic effects of a first volume expansion in hypotensive preterm infants during the first 24 h of life.

**Study design:**

Single-center retrospective observational pilot study including preterm infants ≤31 + 6 weeks of gestation, intubated and mechanically ventilated, presenting arterial hypotension within 24 h of life. All infants received a first volume expansion with modified fluid gelatin (20 mL/kg). Clinical and echocardiographic parameters were compared immediately before and after volume expansion.

**Results:**

Thirty-one infants were included. Volume expansion was associated with a significant increase in systolic, diastolic and mean arterial pressure (median MAP increase: +4 mmHg; +17%), and a significant decrease in heart rate and capillary refill time. Echocardiographic assessment showed a significant increase in left ventricular end-diastolic diameter and superior vena cava flow (median increase: +19%), suggesting improved preload and systemic blood flow. No immediate clinically apparent adverse events were recorded during the observation period.

**Conclusion:**

In this exploratory pilot study, a first volume expansion was associated with short-term improvements in clinical and echocardiographic hemodynamic parameters in hypotensive preterm infants. These findings are hypothesis-generating and cannot be generalized to current filling strategies or repeated fluid boluses.

## 1 Introduction

Hemodynamic disturbances are common in preterm infants during the first hours of life. In clinical practice, this impairment in circulatory adaptation is usually identified by a drop in blood pressure, which tends to decrease in the first hours after birth (1, 2). However, blood pressure measurement is only a component of tissue perfusion, which also depends on cardiac output and heart rate. When these parameters are disturbed, there is a risk of cellular damage by tissue ischemia, followed by ischemia-reperfusion phenomena, particularly at the brain level (3).

Simple clinical parameters used to detect tissue hypoperfusion include blood pressure, urine output, capillary refill time and heart rate. In addition, cardiac ultrasound is a simple, non-invasive method of hemodynamic assessment. It provides a pathophysiological perspective on hemodynamic disturbances and helps guide therapeutic choices. Neonatologists can easily analyze parameters such as shunts through the foramen ovale and ductus arteriosus, ventricular preload, ventricular function and pulmonary pressures (4, 5). Superior vena cava (SVC) flow is another useful parameter, reflecting cerebral perfusion. It has been shown that when SVC flow is below 40 mL/kg/min, there is a clear risk of cerebral hypoperfusion and intraventricular hemorrhage in very premature infants (6, 7).

Treatment of these circulatory adaptation disorders is not standardized. In practice, optimization of ventricular preload by volume expansion is usually proposed as soon as arterial hypotension is present. However, this treatment has not been proven effective on tissue perfusion and can be deleterious. It is relevant in case of hypovolemia, but this situation is not constant, and sometimes a vasopressor or inotropic treatment will be introduced as a first-line therapy (8–11).

Volume expansion fluids are numerous and classified as colloids or crystalloids. There is no official recommendation on the use of one filling solution or another (12). Crystalloids include balanced solutions, ringer lactate and 0.9% NaCl. Colloids include blood-derived products such as concentrated red blood cells, albumin or modified fluid gelatin. Modified fluid gelatin 4 contains 150 mL/L sodium and an osmolarity of 295 mOsm/Kg and has been used as an alternative to crystalloids in case of hypovolemic or vasoplegic shock (13).

Decisions regarding volume expansion should integrate clinical, biological, and ultrasound criteria.

Unwarranted volume expansion can lead to complications such as pulmonary hemorrhage, increased pulmonary shunt through PDA and foramen ovale, or excessive preload leading to pulmonary edema, Moreover, volume overload can contribute to cerebral hemorrhage due to elevated venous pressure, systolic and/or diastolic dysfunction, or impaired cerebral autoregulation (14, 15).

The aim of this study was to describe the clinical and ultrasound the effects of volume expansion in the hypotensive preterm neonate in the first 24 h of life.

The clinical and echocardiographic impact, as well as short- and long-term complications, were analyzed.

The primary outcome was the change in clinical and echocardiographic parameters following volume expansion.

Secondary outcomes included the need for additional hemodynamic support (additional volume expansion or vasopressor therapy) and short- and medium-term neonatal outcomes, including bronchopulmonary dysplasia, intraventricular hemorrhage, retinopathy of prematurity, necrotizing enterocolitis, patent ductus arteriosus treatment, and mortality.

## 2 Material and method

### 2.1 Study design

This was a single-center, retrospective, observational pilot study conducted in the neonatal intensive care unit (NICU) of the Nancy Regional Maternity Hospital (CHRU Nancy, France). The objective was to describe the short-term clinical and echocardiographic effects of a first volume expansion in hypotensive preterm infants during the first 24 h of life.

### 2.2 Study population

Preterm infants born between 26 weeks and 31 weeks + 6 days of gestation between June 29, 2010, and December 17, 2010, and admitted to the NICU during the study period were eligible for inclusion.

Inclusion criteria were: invasive mechanical ventilation, arterial hypotension within the first 24 h of life, defined as a mean arterial pressure (MAP) lower than gestational age in completed weeks, performance of a targeted neonatal echocardiographic assessment, administration of a first volume expansion with modified fluid gelatin.

Only intubated infants were included, as they represented the most severely ill population at risk of early hemodynamic instability during the study period.

Exclusion criteria were: congenital heart disease other than patent ductus arteriosus, patent foramen ovale, or a small ventricular septal defect (<2 mm), ventricular systolic dysfunction on echocardiography, chromosomal abnormalities, prior hemodynamic treatment before echocardiographic assessment, including volume expansion or vasopressor/inotropic therapy, missing or incomplete echocardiographic or clinical data.

By excluding infants who had already received hemodynamic treatment, the study focused specifically on the physiological response to a first volume expansion.

### 2.3 Data collection

Clinical and echocardiographic data were collected retrospectively from medical records, including electronic and paper charts. Clinical parameters collected immediately before and after volume expansion included: systolic, diastolic, and mean arterial blood pressure, heart rate, body temperature, capillary refill time (CRT). Blood pressure was measured using an oscillometric device with an appropriately sized cuff. For each time point, three consecutive measurements spaced five minutes apart were performed, and the median value was retained for analysis. CRT was assessed by trained neonatal staff using standardized clinical practice.

### 2.4 Echocardiographic assessment

Echocardiographic examinations were performed using an ALOKA® ultrasound system equipped with a 7-MHz phased-array transducer. All examinations were performed by operators experienced in targeted neonatal echocardiography, following a standardized acquisition protocol.

The echocardiographic assessment included: cardiac anatomy and exclusion of structural heart disease, ductus arteriosus: diameter, direction of shunt, and maximal shunt velocity (suprasternal view), foramen ovale: size, shunt direction, and velocity (subcostal view), left ventricular ejection fraction (parasternal short-axis view), left ventricular end-diastolic diameter (LVEDD) measured in parasternal short-axis view, inferior vena cava (IVC) diameter and respiratory variability index measured in longitudinal view at the junction with the right atrium,superior vena cava (SVC) diameter (high parasternal long-axis view) and mean flow velocity (subcostal view). SVC flow was calculated using the following formula: SVC flow (mL/kg/min) = mean velocity (cm/s) × π × (mean radius in cm)^2^ × 60/body weight (kg). Mean values were calculated from at least two consecutive measurements, averaged over multiple cardiac cycles when feasible. Pulmonary arterial pressure was estimated when possible using tricuspid regurgitation velocity and ductal shunt characteristics (Supplementary Annexe 1).

### 2.5 Volume expansion protocol

All infants received a first volume expansion with modified fluid gelatin at a dose of 20 mL/kg administered over 1 h, according to the local protocol in use during the study period.

Echocardiographic and clinical assessments were performed immediately before volume expansion and immediately after completion of the infusion.

The use of modified fluid gelatin reflects local practice at the time of inclusion and does not represent current standard recommendations.

### 2.6 Statistical analysis

Categorical variables are presented as numbers and percentages. Continuous variables are presented as medians with interquartile ranges (IQR).

Comparisons between pre- and post-volume expansion values were performed using the Wilcoxon signed-rank test for paired data. A *p*-value < 0.05 was considered statistically significant.

Statistical analyses were performed using SAS/STAT software (version 9.4, SAS Institute, Cary, NC, USA). The statistical analysis was conducted in collaboration with experienced statisticians.

A *post hoc* exploratory analysis was conducted to assess the proportion of infants with a ≥ 10% and ≥15% increase in SVC flow following volume expansion. These thresholds were selected pragmatically to reflect commonly accepted limits of measurement variability in echocardiography.

## 3 Results

### 3.1 Study population

During the study period, 78 preterm infants born between 26 and 31 weeks + 6 days of gestation were admitted to the NICU. Among them, 71 infants required invasive mechanical ventilation on day 0. Forty-seven ventilated infants presented arterial hypotension within the first 24 h of life.

**Figure 1:**
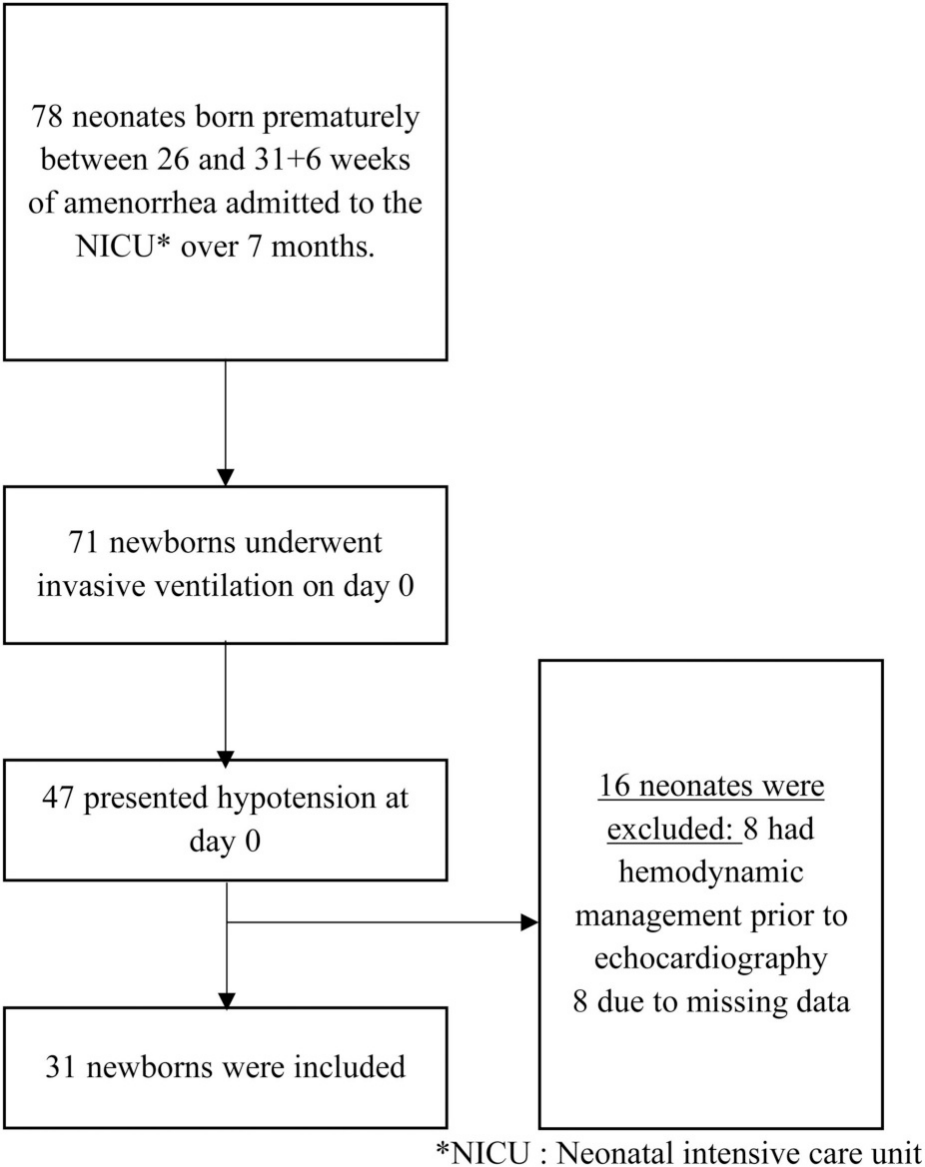
Flow chart. *NICU, neonatal intensive care unit.

Thirty-one infants met all inclusion criteria and were included in the analysis. Sixteen infants were excluded: eight because they had received hemodynamic treatment before echocardiographic assessment, and eight due to missing clinical or echocardiographic data (Figure 1).

The median gestational age of included infants was 29 weeks (IQR 28–30), and the median birth weight was 1,238 g (IQR 1,218–1,254). The median age at inclusion was 3 h of life (IQR 2–5). Baseline characteristics of the population are presented in Table 1.

**Table 1:** Characteristics of the population.

Variable	Median(Q1; Q3)	*N* (%)
Gestational age (week of amenorrhea)	29 (28; 30)	
Weight (g)	1,238 (1,218; 1,254)	
Apgar 1 min	5 (3; 6.5)	
Apgar 5 min	7 (6; 8)	
pH at the umbilical cord	7.295 (7.26; 7.33)	
Lactic acid at the umbilical cord (mmol/L)	2.9 (2.3; 4.7)	
IUGR^a^		3 (10%)
Premature rupture of membranes		12 (39%)
Cesarean section		20 (65%)
General anesthesia		6 (20%)
Peridural anesthesia		21 (70%)
Hyaline membrane disease		19 (61%)

Values are presented as median (IQR) or *N* (% of 31 infants).

^a^ IUGR, intrauterine growth restriction.

### 3.2 Clinical effects of volume expansion

Following volume expansion, significant changes were observed in all measured clinical parameters (Table 2).

**Table 2:** Effect of volume expansion on clinical parameters.

Parameter	Before [median (Q1; Q3)]	After [median (Q1; Q3)]	*p*-value
SBP^a^ (mmHg)	37 (34.5; 40)	43 (39.5; 47)	<0.0001
DBP^b^ (mmHg)	17 (15; 19.5)	20 (16.5; 24.5)	<0.0001
MBP^c^ (mmHg)	24 (23; 26)	28 (24.5; 31)	<0.0001
HR^d^ (bpm)	155 (144; 171)	145 (136.5; 149)	<0.0001
CRT^e^ (s)	2.5 (2; 3)	2 (1.5; 2.2)	0.0019

^a^ SBP, systolic blood pressure.

^b^ DBP, diastoli cblood pressure.

^c^ MBP, mean blood pressure.

^d^ HR, heart rate; CRT, capillary refill time.

^e^ Capillary refill time (CRT).

Median systolic blood pressure increased from 37 mmHg (IQR 34.5–40) to 43 mmHg (IQR 39.5–47) (*p* < 0.0001). Median diastolic blood pressure increased from 17 mmHg (IQR 15–19.5) to 20 mmHg (IQR 16.5–24.5) (*p* < 0.0001). Median mean arterial pressure increased from 24 mmHg (IQR 23–26) to 28 mmHg (IQR 24.5–31), corresponding to a median relative increase of 17% (*p* < 0.0001).

Heart rate significantly decreased from a median of 155 bpm (IQR 144–171) to 145 bpm (IQR 136.5–149) (*p* < 0.0001). Capillary refill time decreased from 2.5 s (IQR 2–3) to 2 s (IQR 1.5–2.2) (*p* = 0.002).

### 3.3 Echocardiographic effects of volume expansion

Echocardiographic parameters before and after volume expansion are summarized in Table 3.

**Table 3:** Effect of volume expansion on echocardiographic parameters.

Parameter	Before [Median (Q1; Q3) or *N* (%)]	*N*	After [Median (Q1; Q3) or *N* (%)]	*N*	*p*-value
PDA^a^					
Yes	30 (96%)	31	30 (100%)	30	
Diameter (mm)	1.8 (1.3; 2.45)	30	1.5 (1.15; 2.3)	30	0.13
Shunt direction	11 (37%)	30	12 (40%)	30	1
LR^b^ Bidirectionnal	19 (63%)		18 (60%)		
Shunt LR velocity (m/s)	0.86 (0.55; 1.26)	30	1.07 (0.8; 1.46)	30	0.004
Shunt RL^c^ velocity (m/s)	0.34 (0–0.64)	19	0.39 (0; 0.78)	18	0.9
Right-left time to cycle ratio	0.16 (0; 0,285)	19	0.17 (0; 0.3)	18	0.63
Systemic pulmonary arterial pressure (mmhg)	34 (31; 38.75)	30	37 (34; 41.7)	30	0.05
TR^d^					
Systemic pulmonary arterial pressure (mmhg)	29 (23.5; 33)	19	33 (30; 36)	21	0.07
PFO^e^					
Yes	26 (84%)	31	28 (93%)	30	
Measure (mm)	2,3 (1.6; 2.9)	25	2.5 (1.5; 3.4)	28	0.33
LR shunt	5 (20%)	25	12 (43%)	28	
Bidirectionnal shunt	20 (80%)	25	16 (57%)	28	
Shunt LR velocity (m/s)	0.36 (0.2; 0.55)	20	0.57 (0.48; 0.77)	12	0.81
Shunt RL velocity (m/s)	0.16 (0; 0.225)	28	0.12 (0; 0.2)	24	0.02
LVEF^f^ (%)	75 (71.5; 80)	30	75.5 (70.7; 79.25)	29	0.8
Left ventricular end-diastolic diameter (mm)	11.6 (11.05; 12.8)	30	12.9 (11.9; 13.6)	29	<0.01
Mean velocity of left pulmonary artery (m/s)	0.35 (0.3; 0.42)	29	0.44 (0.37; 0.47)	28	0.01
Inferior vena cava					
Variability index	0.25 (0.13–0.35)	30	0.20 (0.09; 0.4)	29	0.59
Superior vena cava					
Max. diameter (mm)	3.4 (2.8; 3.85)	28	4 (3.2; 4.45)	23	0.001
Mean velocity (m/s)	0.20 (0.15; 0,25)	26	0.20 (0.16; 0.26)	28	0.74
Output (mL/kg/min)	82.8 (48.8; 104.1)	24	99 (66.3; 99)	23	0.02

^a^ PDA, patent ductus arteriosus.

^b^ LR, left to right.

^c^ RL, right to left.

^d^ TR, tricuspide regurgitation.

^e^ PFO, patent foramen ovale.

^f^ LVEF, left ventricular ejection fraction.

Left ventricular end-diastolic diameter significantly increased from a median of 11.6 mm (IQR 11.05–12.8) to 12.9 mm (IQR 11.9–13.6) (*p* < 0.01), suggesting increased ventricular preload. Superior vena cava diameter increased significantly, and SVC flow increased from a median of 82.8 mL/kg/min (IQR 48.8–104.1) to 99 mL/kg/min (IQR 66.3–99), corresponding to a median relative increase of 19% (*p* = 0.02). Mean SVC flow velocity did not change significantly.

Although median SVC flow increased at the group level, individual responses were heterogeneous. In a *post hoc* exploratory analysis, 13 of 22 infants (59.1%, 95% CI 38.7–76.7%) exhibited a ≥ 10% increase in SVC flow after volume expansion. A ≥ 15% threshold yielded comparable findings (54.5%).

The velocity of the left-to-right ductal shunt increased significantly after volume expansion (*p* = 0.004), whereas ductal diameter and shunt direction did not differ significantly. No significant changes were observed in left ventricular ejection fraction.

Inferior vena cava diameter and respiratory variability index did not significantly change after volume expansion.

### 3.4 Subsequent hemodynamic management and outcomes

Following the first volume expansion, 15 infants (48%) required additional hemodynamic support. All of these infants received vasopressor therapy with dopamine and/or dobutamine. None received hydrocortisone during the study period.

Short- and medium-term neonatal outcomes are summarized in Table 4. No immediate clinically apparent adverse events were recorded during the study observation period. Given the exploratory design and limited sample size, no comparative analysis was performed between infants requiring additional hemodynamic support and those who did not.

**Table 4:** Complications by patients treated.

Parameter	*N* (%)
BPD^a^	16 (53%)
Moderate BPD	12 (40%)
Mid BPD	2 (6.5%)
Severe BPD	2 (6.5%)
PDA tretaed by ibuprophen	3 (10%)
PDA treated by surgery	2 (6%)
IVH^b^	4 (13%)
IVH I	3 (10%)
IVH II	0
IVH III	1 (3%)
IVH IV	0
Retinopathy	2 (7%)
Ulceronecrotizing enterocolitis	3 (10%)
Death	1 (3%)

^a^ BPD, bronchopulmonary dysplasia.

^b^ IVH, intraventricular haemorrhage.

## 4 Discussion

In this pilot observational study, we describe the short-term clinical and echocardiographic effects of a first volume expansion in hypotensive preterm infants during the first 24 h of life. Volume expansion with modified fluid gelatin was associated with an increase in arterial blood pressure, a decrease in heart rate, and changes in echocardiographic parameters suggesting improved preload and systemic blood flow.

The observed increase in systolic, diastolic and mean arterial pressure following volume expansion is consistent with previous reports describing transient blood pressure improvement after fluid administration in preterm infants (1, 2, 10). However, blood pressure alone is an incomplete surrogate of tissue perfusion in neonates. Tissue perfusion depends not only on arterial pressure but also on cardiac output and vascular resistance, and should therefore be interpreted in a broader hemodynamic context (3, 8, 16).

Heart rate significantly decreased after volume expansion. Since cardiac output is the product of heart rate and stroke volume, tachycardia may represent a compensatory mechanism in response to reduced stroke volume during hypovolemia (3). The decrease in heart rate observed after volume expansion may therefore reflect improved ventricular filling. However, heart rate is influenced by multiple factors in preterm infants, including pain, temperature, sedation and respiratory status, and should not be interpreted in isolation (17).

From an echocardiographic perspective, the significant increase in left ventricular end-diastolic diameter (LVEDD) observed after volume expansion suggests an increase in ventricular preload. LVEDD is commonly used as a marker of preload but remains dependent on loading conditions and operator expertise (18). In the present study, stroke volume and cardiac output were not directly measured using left ventricular outflow tract velocity–time integral. Although these parameters are widely used to define fluid responsiveness, their interpretation may be challenging in the early neonatal period due to frequent ductal shunting, which alters the relationship between left ventricular output and systemic blood flow (5, 18). This represents an important limitation of our study.

Superior vena cava (SVC) flow significantly increased following volume expansion. SVC flow has been proposed as a surrogate marker of systemic blood flow and cerebral perfusion in preterm infants, and low SVC flow has been associated with adverse neurological outcomes, including intraventricular hemorrhage (5–7, 19). However, SVC flow measurement is technically demanding and subject to significant variability related to respiratory cycle, ventilation mode, shunt physiology and Doppler alignment (20, 21). As such, SVC flow should be interpreted cautiously and integrated into a multimodal hemodynamic assessment rather than used as a standalone parameter.

Inferior vena cava (IVC) diameter and variability index did not significantly change after volume expansion. IVC measurements are frequently used to estimate preload in spontaneously breathing patients, but their reliability is reduced under positive pressure ventilation (21). In our cohort, all infants were intubated and mechanically ventilated, which may explain the absence of significant changes in IVC parameters. This may differ in contemporary neonatal practice, where non-invasive ventilation is more frequently used, partly due to the introduction of early low-dose hydrocortisone as demonstrated in the PREMILOC trial (22).

Capillary refill time (CRT) improved after volume expansion; however, no consistent relationship was observed between CRT and echocardiographic parameters. CRT is a simple bedside marker of peripheral perfusion but remains subjective and difficult to standardize in preterm infants (20). Previous studies have shown poor correlation between CRT and systemic blood flow measurements, including SVC flow (21, 23). Therefore, CRT should be considered as one component of a global hemodynamic assessment rather than a reliable marker of response to volume expansion.

Importantly, infants who had received prior hemodynamic treatment were excluded from this study. Consequently, our findings are only applicable to the first volume expansion. Fluid responsiveness is known to decrease with repeated boluses as patients move along the flat portion of the Frank–Starling curve (17). Our results cannot be extrapolated to repeated volume expansion or more advanced stages of circulatory failure.

No early or late complications directly attributable to volume expansion were observed in this cohort. However, this study was not designed nor powered to assess safety outcomes. Excessive volume expansion has previously been associated with adverse outcomes in preterm infants, including pulmonary hemorrhage and increased mortality (14, 15). Moreover, the filling strategy used in this study—modified fluid gelatin—is no longer recommended in current practice, where crystalloids are generally preferred (13, 14). This choice reflects local practice at the time of inclusion and constitutes a major limitation regarding the generalizability of our findings.

This study has several limitations. Its retrospective and single-center design, small sample size, absence of a control group, and use of a volume expansion fluid no longer routinely used limit causal inference and external validity. The lack of direct stroke volume measurement and limited assessment of biological markers of perfusion further restrict physiological interpretation. Nevertheless, in the context of early hypotension, the inclusion of a control group would raise ethical concerns (10).

This study was conducted in a different era of neonatal intensive care, before the widespread use of contemporary ventilation strategies, standardized sedation protocols, and point-of-care ultrasound. As such, the external validity of our findings to current practice is limited.

However, we believe that these data remain of interest as a physiological description of the heterogeneous hemodynamic response to volume expansion in preterm infants, highlighting that hypotension does not systematically imply preload dependency.

Our study was not designed to systematically assess adverse effects of volume expansion. Ventilatory parameters and detailed echocardiographic shunt dynamics were not prospectively collected after fluid administration. Therefore, subtle or delayed complications such as increased pulmonary shunting or fluid overload cannot be excluded. In addition, cumulative exposure to repeated fluid boluses, which may contribute to fluid-related morbidity, was not addressed by our study design.

Despite these limitations, our study has notable strengths. Echocardiographic assessments were performed by experienced operators using standardized protocols and the same ultrasound equipment. The paired before–after design allowed each infant to serve as their own control, reducing inter-individual variability. These pilot data provide useful information for the design and power calculation of future prospective studies evaluating contemporary volume expansion strategies integrated with multimodal hemodynamic assessment in preterm infants.

## 5 Conclusion

This pilot observational study describes the short-term clinical and echocardiographic effects of a first volume expansion in hypotensive preterm infants during the first 24 h of life. Volume expansion was associated with transient improvements in arterial blood pressure, heart rate, and echocardiographic markers of preload and systemic blood flow.

Given the exploratory nature of the study, the limited sample size, and the use of a filling strategy that does not reflect current practice, these results should be interpreted with caution. They do not allow firm conclusions regarding efficacy, safety, or fluid responsiveness, nor can they be extrapolated to repeated volume expansions or other filling solutions.

Our findings provide preliminary data that may help inform the design and power calculation of future prospective studies evaluating contemporary volume expansion strategies integrated with multimodal hemodynamic assessment in preterm infants.

## Data Availability

The raw data supporting the conclusions of this article will be made available by the authors, without undue reservation.
