# Supplementary material for: Clinical and echocardiographic response to volume expansion in hypotensive preterm infants: a pilot observational study
Source: Front Pediatr. 2026 Mar 4;14:1749902. doi: 10.3389/fped.2026.1749902 (PMC12996099; doi:10.3389/fped.2026.1749902)
Supplement: Supplementary file 1 [file Datasheet1.doc]

**FICHE ETUDE ECHOFILLING (1)**

**Fiche N°** |__|__|__|

NOM : |_______| Prénom : |_______| Date naissance : |__|__||__|__||__|__| Heure : |__|__| h AG : |__|__| SA|__|j

Poids naissance : |__|__|__|__| g

Traitement maternel < 48 h : OUI ❒ NON ❒

Antibiotiques : ❒ (_______) Adalate : ❒, Aldomet : ❒, Loxen ❒ , Sulfate Mag ❒, Trandate : ❒, Autre : ❒ …………………….

Maturation : OUI ❒ NON ❒ Nombre de cures : |__|__| RPM : OUI ❒ NON ❒ Nbre de jours : |__|__|

Accouchement : VB ❒ Césarienne : ❒ Apgar : |__|__| / |__|__| pH au cordon : |__|__| Lactate au cordon : |__|__|

Mode V : VMC ❒ OHF ❒ FiO2 au moment inclusion : |__|__| % PM au moment inclusion : |__|__|

MMH : OUI ❒ NON Surfactant ❒OUI ❒ NON ❒ Nombre de doses au moment inclusion : |__|__|

Sédatif : OUI ❒ NON ❒ Hypnovel : ❒ Sufenta : ❒ (préciser moment, posologie et mode d’administration)

**CRITERES D’INCLUSION**

BB intubé ❒ AG ≤ 30 SA ❒ Age < 24 h ❒ Hypotension ❒

**CRITERES D’EXCLUSION**

Cardiopathie ❒ Syndrome polymalformatif ❒ Anomalie chromosomique ❒

Traitement à visée hémodynamique avant inclusion ❒ Remplissage ❒ Amines ❒

**Nombre d’heures de vie au moment de l’inclusion** : |__|__|

**Paramètres hémodynamiques AVANT remplissage Paramètres hémodynamiques APRES remplissage**

|  | PAS | PAD | PAM | FC | T° | TRC |  |  | PAS | PAD | PAM | FC | T° | TRC |
| --- | --- | --- | --- | --- | --- | --- | --- | --- | --- | --- | --- | --- | --- | --- |
| M-10 |  |  |  |  | ///// |  |  | M1 |  |  |  |  | ///// |  |
| M-5 |  |  |  |  | ///// |  |  | M1+5 |  |  |  |  | ///// |  |
| M 0 |  |  |  |  |  |  |  | M1+10 |  |  |  |  |  |  |

**ECHO cœur N°1**

**CA** : OUI ❒ NON ❒, Taille : |__|__| mm, shunt : D/G

G/D ❒ et BiD ❒ alors temps DG/tps du cycle : 0,|__|__|

Vélocité : |__|__| cm/s PAPS 1 : |__|__| mmHg

Vélocité moyenne APG : |__|__|cm/s

**FOP** : OUI ❒ NON Taille : |__|__| mm

Direction shunt FOP : D/G ❒, G/D ❒ (gradient OG/OD|__|__| mmHG), BiD ❒

**IT** : OUI ❒ NON ❒

Vélocité : |__|__| cm/s PAPS2 : |__|__| mmHg

**FE** : |__|__| % **DTDVG** : |__|__| mm

**VCI** :

|  | Mesure 1 | Mesure 2 | Moyenne |
| --- | --- | --- | --- |
| d. max |  |  |  |
| d. min |  |  |  |

Index de variabilité de la VCI : |__|__|__| %

**Débit VCS**:

|  | Mesure 1 | Mesure 2 | moyenne |
| --- | --- | --- | --- |
| d. max |  |  |  |
| d. mini |  |  |  |
| Vel moy |  |  |  |
| Débit | //////////// | //////////// |  |

**ECHO cœur N°2**

**CA** : OUI ❒ NON ❒, Taille : |__|__| mm, shunt : D/G

G/D ❒ et BiD ❒ alors temps DG/tps du cycle : 0,|__|__|

Vélocité |__|__| cm/s PAPS 1 : |__|__| mmHg

Vélocité moyenne APG |__|__|

**FOP** : OUI ❒ NON Taille : |__|__| mm

Direction shunt FOP : D/G ❒, G/D ❒ (gradient OG/OD|__|__| mmHG), BiD ❒

**IT**: OUI ❒ NON ❒

**Vélocité** |__|__| cm/s PAPS2 : |__|__| mmHg

**FE** |__|__| % **DTDVG** : |__|__| mm

**VCI** :

|  | Mesure 1 | Mesure 2 | Moyenne |
| --- | --- | --- | --- |
| d. max |  |  |  |
| d. min |  |  |  |

Index de variabilité de la VCI : |__|__|__| %

**Débit VCS** :

|  | Mesure 1 | Mesure 2 | moyenne |
| --- | --- | --- | --- |
| d. max |  |  |  |
| d. mini |  |  |  |
| Vel moy |  |  |  |
| Débit | //////////// | //////////// |  |

**FICHE ETUDE ECHOFILLING (2)**

**Fiche N°** |__|__|__|

**evolution**

**1 - Court terme (dans les 48h qui suivent l’inclusion)**

Age extubation  : |__|__| jours

Remplissages  ultérieurs: OUI ❒ NON ❒ Quantité |__|__|__| ml/kg

Amines : OUI ❒ NON ❒ si OUI : Dopamine ❒ dose max |__|__| durée t |__|__| j

Dobutamine ❒ dose max |__|__| durée t |__|__| j

Autres ❒ dose max |__|__| durée t |__|__| j

NOi : OUI ❒ NON ❒

Surfactant (après écho 2) : OUI ❒ NON ❒ Nombre doses : |__|__|

**2 – MOYEN TERME : (jusqu’à la sortie de l’enfant)**

DBP : OUI ❒ NON ❒

O2 J28 de vie : OUI ❒ NON ❒ Si OUI : FiO2 = |__|__| %

O2 J36 SA APC : OUI ❒ NON ❒ Si OUI : FiO2 = |__|__| %

PCA : OUI ❒ NON ❒ Ibuprofène : OUI ❒ NON ❒ Nbre cures : |__|__|

Trait^t^ chirurgical : OUI ❒ NON ❒

HIV * : OUI ❒ NON ❒ Si OUI : stade : ……………………………..

Rétinopathie * : OUI ❒ NON ❒ Si OUI : stade : ……………………………..

ECUN * : OUI ❒ NON ❒ Si OUI : stade : |__|

Survenue décès : OUI ❒ NON ❒ Si OUI : âge du décès : |__|__| j

Cause directe décès :

❒ arrêt des soins pour lésions neurologiques

❒ insuffisance respiratoire terminale

❒ infection

❒ ECUN

❒ Autre : préciser :
